# Structured Light-Based 3D Reconstruction System for Plants

**DOI:** 10.3390/s150818587

**Published:** 2015-07-29

**Authors:** Thuy Tuong Nguyen, David C. Slaughter, Nelson Max, Julin N. Maloof, Neelima Sinha

**Affiliations:** 1 Department of Computer Science, University of California, Davis, CA 95616, USA; E-Mail: max@cs.ucdavis.edu; 2 Department of Biological and Agricultural Engineering, University of California, Davis, CA 95616, USA; 3 Department of Plant Biology, University of California, Davis, CA 95616, USA; E-Mails: jnmaloof@ucdavis.edu (J.N.M.); nrsinha@ucdavis.edu (N.S.)

**Keywords:** plant phenotyping, 3D reconstruction, stereo vision, structured light, point cloud, 3D feature extraction

## Abstract

Camera-based 3D reconstruction of physical objects is one of the most popular computer vision trends in recent years. Many systems have been built to model different real-world subjects, but there is lack of a completely robust system for plants.This paper presents a full 3D reconstruction system that incorporates both hardware structures (including the proposed structured light system to enhance textures on object surfaces) and software algorithms (including the proposed 3D point cloud registration and plant feature measurement). This paper demonstrates the ability to produce 3D models of whole plants created from multiple pairs of stereo images taken at different viewing angles, without the need to destructively cut away any parts of a plant. The ability to accurately predict phenotyping features, such as the number of leaves, plant height, leaf size and internode distances, is also demonstrated. Experimental results show that, for plants having a range of leaf sizes and a distance between leaves appropriate for the hardware design, the algorithms successfully predict phenotyping features in the target crops, with a recall of 0.97 and a precision of 0.89 for leaf detection and less than a 13-mm error for plant size, leaf size and internode distance.

## 1. Introduction

Automation is necessary in the agricultural industry to help accelerate the rate of increased crop productivity through genetic improvement techniques, in order to help cope with the rapid increase in human population and future demands on worldwide food security. Phenotyping of new and old varieties under varying environmental conditions to assess their suitability presents a challenge [1,2]. There is a need for developing novel, field-deployable systems with semi- or fully-automatic processing of plant phenotypes for a suite of vegetative traits that can aid in our understanding of the relationships between genetic information and food productivity. The critical elements of such systems are the sensors that help to automate phenotyping and contribute knowledge to the final understanding of this complex relationship. Most of the sensors used in agriculture have limited resolution or dimensionality and are not able to acquire the full scope of available information about plants, such as their structure and leaf texture. This leads to a limitation in distinguishing different types of deficiencies [3]. Advanced sensors, like cameras, that can characterize spatial and color information from natural objects will play a crucial role in the future development of agricultural automation [4,5].

Recent methods for sensor-based 3D reconstruction have been developed for a wide range of applications. A method for 3D shape scanning with a time-of-flight (ToF) camera has been described in [6]. The ToF camera technique can measure depth information in real-time, and when the method [6] is used to align depth scans in combination with a super-resolution approach, some mitigation of the sensor’s typically high signal noise level and systematic bias can be achieved. In [7], a “visual structure-from-motion system” for 3D reconstruction is implemented via feature extraction, image matching and dense reconstruction algorithms. This structure-from-motion implementation works successfully on rigid, enclosed (*i.e.*, non-porous) objects, with the use of a single camera capturing images from multiple viewpoints. A stereo vision technique applied to 3D reconstruction is introduced in [8]. This work provides a framework to create visually realistic 3D reconstructions of solid objects, including the steps of stereo image acquisition, feature detection, feature matching, camera calibration matrix calculation, point cloud generation and surface reconstruction. A stereo vision-based 3D reconstruction system for underwater scenes is proposed in [9]. This system yields promising depth map results in exploring underwater environments. There is a great deal of work that utilizes consumer-grade range camera technology (e.g., the Microsoft Kinect) [10] for scene reconstruction. The Kinect, which works in a similar way to a stereo camera, was originally designed for indoor video games. Due to its robustness and popularity, it is being studied for use in many research and industrial applications. In [11], the Kinect device was utilized to reconstruct dense indoor scenes.

A number of stereo vision-based methods have been developed for 3D modeling of plants and leaves. The research in [12] presents a combination of binocular stereo vision and structure-from-motion techniques [7] for reconstruction of small plants from multiple views. The 3D reconstruction results for the plant canopy include height, width and volume of the plant, as well as leaf cover area. In [13], color and ToF cameras were used to model leaves through a combination of hierarchical color segmentation and quadratic surface fitting using ToF depth information. A moving robot arm holding a camera was additionally employed to inspect the quality of leaf segmentation. The study [14] shows how the stereo and ToF images can be combined for non-destructive automatic leaf area measurements. In [15], the Microsoft Kinect was utilized in combination with the Point Cloud Library [16] to provide measures of plant height and base diameter in “tower mode”. In [17], Kinect-based visualization of potted greenhouse tomato plants in indoor environments is presented with a method of automatic plant stem detection. The work in [18] is mostly similar to [15] with the addition of depth information-based leaf segmentation. A novel 3D mesh-based technique was presented in [19] for temporal high-throughput plant phenomics. Based on the plant meshes reconstructed using commercial software for 3D scanning [20], this study provided mesh segmentation, phenotypic parameter estimation and plant organ tracking over time to yield promising measurement accuracies of stem height and leaf size. In [21], the Kinect sensor was assessed to determine its best viewing angles to estimate the plant biomass based on poplar seedling geometry. The purpose of [22] is to analyze the accuracy of a structure-from-motion combined with the multiview stereo method for tomato plant phenotyping at the organ level, based on the reference data provided by a close-up laser scanner. The extracted 3D features herein include leaf area, main stem height and convex hull of the complete plant. In [23], the first steps of utilizing 3D light field cameras were introduced for phenotyping large tomato plants in a greenhouse environment. Promising results of 3D reconstructed greenhouse scenes were shown in this work with several aspects related to camera calibration, lens aperture, flash illumination and the limitation of the field of view. There are recent studies that use 3D laser scanning for plant phenotyping. In [24], a surface feature histogram-based approach is introduced to adapt to laser scans of plants for the purpose of plant organ classification. Local geometric point features are used to describe the characteristics of plant organ classes. Classification results of grapevine and wheat organs are shown with very high reliability in this study. High throughput phenotyping of barley organs based on 3D laser scanning is presented in [25]. By combining the advantages of a surface feature histogram-based approach with a parametric modeling of plant organs, this work shows automatic parameter tracking of the leaves and stem of a barley plant over time.

This paper describes a novel 3D reconstruction system for plants that incorporates a number of unique hardware-based stereo features: multiple pairs of high-resolution color digital cameras, visible structured lights, ease of configuration adjustment and the ability to work indoors and outdoors. There are three principal contributions of this research. First, a custom mechanical structure for multi-view 3D reconstruction of plants was designed that consists of an arc to hold ten high-resolution digital color cameras, a plant-stationary mount for two visible structured lights and a target turn-table for a 360-degree view of the plant. Second, a custom design for structured lights was created that projects random-dot patterns onto the target to enhance the uniqueness of the visual texture on the object surface, so that significantly better stereo matching results can be obtained. Computer control of illumination synchronization and intensity allows the system to adapt to indoor and outdoor scenes. Third, a complete hardware and software solution (including camera calibration, structured light control, stereo matching, the proposed 3D point cloud generation and registration, point cloud noise removal and segmentation and the proposed 3D leaf detection and 3D plant feature measurement) is created for both 3D reconstruction and non-destructive phenotyping measurement (plant height, number of leaves, leaf height and width and internode distances) of plants.

## 2. System Design

**Figure 1:**
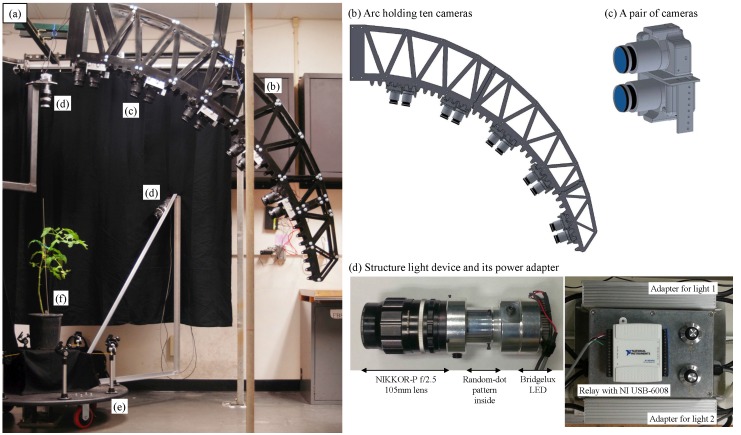
Mechanical structure (**a**) of the 3D reconstruction system: (**b**) the arc holding ten Canon EOS Rebel T3 cameras; (**c**) a pair of cameras where the second camera is upside down relative to the first one; (**d**) structured light devices and their power adapters with a relay controlled by a digital I/O control NI USB-6008; (**e**) a turn-table that rotates the plant 360 degrees; and (**f**) the target plant.

The 3D image data were based on digital color images of individual plants taken by ten electronically-controlled, high-resolution, digital single-lens reflex cameras (Model EOS Rebel T3, Canon Inc., Tokyo, Japan). The ten cameras, each equipped with a zoom lens (Model EF-S 18–55 mm 1:3.5–5.6 IS II, Canon Inc., Tokyo, Japan), were organized into five stereo camera pairs aimed at the target object and held fixed relative to one another on an arc at 25, 40, 55, 70 and 85 degrees relative to the ground plane. Figure 1 shows the mechanical structure of our 3D reconstruction system, where Figure 1b shows the arc and cameras. One camera in each stereo pair was mounted “upside down” relative to the other in order to place the lens centerlines on exactly parallel optical axes with their pairwise baseline set to 89 mm. A Universal Serial Bus (USB) hub was used to connect all cameras to a computer and to allow complete control of the cameras by an open-source software application *digiCamControl* [26]. All ten color images, with a resolution 1920 × 1280 pixels, were captured and transferred to the computer at once via the USB. Because a difference in image resolution might dramatically affect the processing speed of the system from image transfer, segmentation and stereo matching to point cloud processing, we selected the resolution of 1920 × 1280 as the best trade-off between speed and quality, so that highly-accurate results can be obtained in an acceptable processing time. Camera parameters, including focal length, aperture, shutter speed, ISO and white balance, were manually set to achieve the best quality images for camera calibration and stereo matching.

A structured illumination system was designed to provide plant-stationary active visual texture enhancement of plant foliage from all camera viewpoints. The system utilized two telephoto 105-mm focal length lenses (Model NIKKOR-P f/2.5, Nikon Co., Tokyo, Japan), each equipped with a high-power, 29 mm-diameter LED array (Model BXRC-40E10K0-L-03, Bridgelux Inc., Livermore, CA, USA, white color, maximum 10,000 luminous flux and correlated color temperature of 4000 K), designed to project two grayscale, random-dot patterns printed in high resolution on clear film onto the scene. The brightness and strobe synchronization of the LED arrays could be controlled by a dimmable LED power supply (Model HLG-120H-42B, MEAN WELL Enterprises Co., Guangzhou, China) configured with a solid state relay connected to the computer via USB through a digital I/O device (Model USB 6008, National Instruments Co., Austin, TX, USA). Figure 1d shows the structure light device and its power adapter.

**Figure 2:**
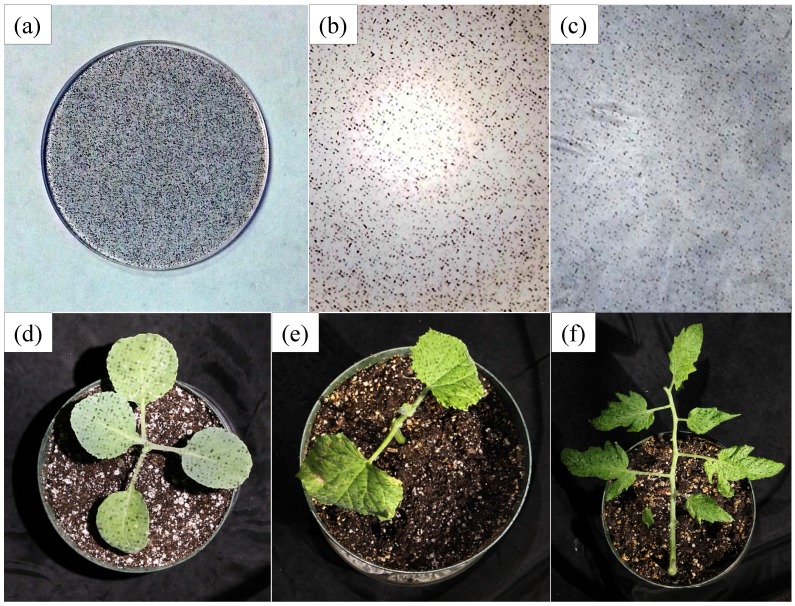
Random-dot pattern projected onto surfaces and plants: (**a**) the dot pattern printed on a transparency film (in this figure, it is under a transparent window); (**b**) an image of the pattern projected (with maximum brightness) on a piece of white paper; (**c**) an image of the pattern projected on a black curtain; (**d**) the pattern projected on a cabbage plant; (**e**) cucumber plant; and (**f**) a compound leaf from a big tomato plant.

For enhanced visual texture generation, a random-dot pattern was created using a 3000 × 3000 pixel grayscale image with a printed resolution of 1200 dots-per-inch, where a 30% Hurl noise was applied to a transparent background. This projected pattern is hence able to support the segmentation and matching algorithms at a three pixel-wide resolution of approximately 1 mm on the leaf surface of a plant. Figure 2 shows the random-dot pattern projected onto white and black surfaces (a piece of paper and a cloth curtain) and plants. The dot pattern, printed on a 38 mm-diameter transparency film (Figure 2a), was placed between two transparent glass windows for support and inserted into the focal plane of the structured light cylinder (Figure 1d). The pattern projection was done with an object distance of approximately 1.5 m to provide good depth of focus. Figure 2b,c shows that even when the object is completely white or black, the structured light can still create added visual texture. In Figure 2d–f, dot textures are created effectively on the leaf surface of the cabbage and cucumber plants and tomato leaves. The two structured light devices were mounted on two arms at a 90-degree top view and a 45-degree side view, respectively. Both of these arms were mounted in a fixed position relative to the plant (Figure 1d,e).

A turn-table was utilized to account for the target plant not being simple, *i.e.*, its leaves are curved or overlapping, causing portions of the plant to be occluded when using a single view angle of the arc relative to the plant. The current choice of optics in the illumination system supports object sizes up to 30 cm × 30 cm × 50 cm.

## 3. Software Algorithms

**Figure 3:**
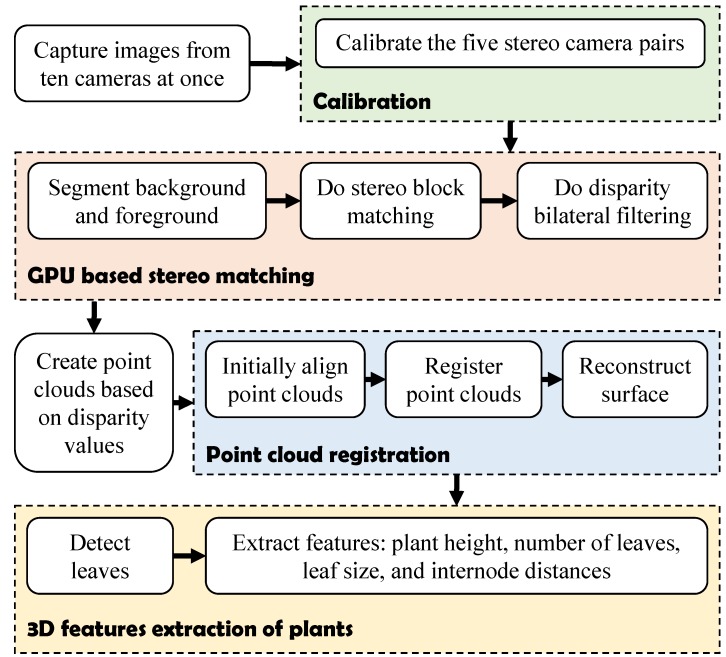
Block diagram representation of the software algorithm.

The algorithm consists of four main stages. First, stereo camera calibration is done after receiving images from the ten cameras. Second, a stereo matching step is performed with GPU (graphics processing unit) accelerated background/foreground segmentation, block matching and disparity bilateral filtering. An intermediate process of reprojecting disparity values to a 3D real-world point cloud is executed after the second step. Third, the output point clouds from the previous stage are merged by a registration algorithm. Afterwards, the final point cloud of a plant is segmented to extract phenotyping features. Figure 3 shows the diagram of the proposed algorithm.

### 3.1. Stereo Camera Calibration

Camera calibration plays a crucial role in many computer vision tasks and is particularly important in stereo vision systems. The precision of the camera calibration directly impacts the accuracy of the estimation of actual object distances. The problem of determining distance information is substantially simplified when an accurate calibration method is applied. In this paper, we utilize the methods in [27,28] and a classical black-white chessboard to calibrate the cameras. Each stereo camera pair, having a baseline of 89 mm, has its own viewpoint of the target plant; we therefore do calibration of the five camera pairs independently. Our chessboard has 54 square-blocks in a 9 × 6 pattern, where each square has a size of 25.4 mm × 25.4 mm. Accurate chessboard size is required to give an accurate estimation of plant features. For the calibration procedure, the chessboard was positioned at 10 different angles in each of the camera pair’s viewpoints so that the system can support plant heights up to 50 cm. Chessboard square corners are detected with subpixel accuracy as the input of the calibration method; and the output includes intrinsic, distortion and extrinsic matrices of the two cameras and the perspective transformation matrix, which will be needed in reprojecting depth information to real-world coordinates.

### 3.2. Stereo Matching

#### 3.2.1. Background and Foreground Segmentation

**Figure 4:**
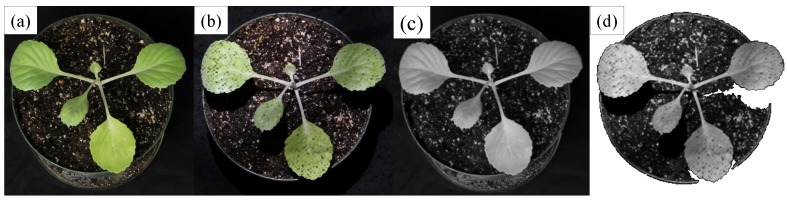
Segmentation of a cabbage plant and its soil from the background. The plant (**a**) without and (**b**) with using structured light; segmentation results of (**c**) without and (**d**) with using structured light; note that (c,d) are the results of binary segmentation in which the plant and its soil are necessarily isolated from the black background; and (c) indicates segmentation failure.

All plant images have to be rectified using the calibration matrices obtained from the previous step to ensure accurate stereo matching performance across all views. The images are then segmented using a GPU-based mean shift segmentation [29,30] to isolate image regions belonging to the target object. Mean shift is a nonparametric iterative algorithm that defines a window of interest around the data points and computes the mean of the data points, then shifts the window center to the mean and repeats the algorithm until it converges. The mean shift segmentation method is a fast and effective technique for this application in which we need to segment the plant from the background, *i.e.*, binary segmentation. This preprocessing step is compatible with the idea of using structured light, where the image background becomes removable while the structured light is running and ambient light in the room is turned off. Figure 4 shows an example of segmenting a cabbage plant (and its soil) with and without using the structured light. We use the same parameter set (the spatial window radius is 11; the color window radius is 7; the minimum segment size is 10; and background threshold is 10) in both cases. When the structured light is used, the plant and soil can be completely isolated from the background (Figure 4d), which helps improve the accuracy of stereo matching. In the case when no structured light is used, the background cannot be separated from the plant and soil; as in Figure 4c, there are low intensity pixels on the background, which the automatic segmentation algorithm cannot accurately assign to the background or object.

#### 3.2.2. Stereo Block Matching

There are currently a number of effective stereo matching algorithms, such as belief propagation [31], census correlation [32] and graph cuts [33]. Here, the focus is on achieving superior matching using an optical mechanism of creating enhanced texture over the object surface. Descriptors, like local binary patterns [34,35], can be used to enhance the texture for stereo matching; however, those descriptors originate from the image itself, *i.e.*, there is no additional support information from the surrounding environment. The proposed structured light physically adds dot texture to the scene so that the structured light-based matching result is significantly improved when applying the same stereo matching algorithm. Considering the trade-off between accuracy and processing time, we utilize GPU-based block matching for fast processing speed, giving high quality results with the structured light.

#### 3.2.3. Disparity Bilateral Filtering

For further improvement of the quality of depth information, an effective joint bilateral filter is proposed in [36] to preserve the disparity discontinuity, *i.e.*, to maintain the object’s edge. This filter works under the assumption that color information is a high-weight factor in determining the disparity discontinuity. Defining the disparity map obtained from the block matching algorithm to be *D*, the reference image (the original image from the left camera was the reference for this enhancement step) to be $I_{r}$ and the window radius of the bilateral filter to be *r*. At every pixel p={x,y}, we define:

$$\begin{array}{ccc} {\vec{d}}_{p} & = & \left\{D(x-1,y),D(x,y-1),D(x+1,y),D(x,y+1)\right\}\\ {\vec{u}}_{p}^{x} & = & \left\{x-r,x-(r-1),...,x+(r-1),x+r\right\}\\ {\vec{u}}_{p}^{y} & = & \left\{y-r,y-(r-1),...,y+(r-1),y+r\right\} \end{array}$$

then the disparity map can be updated sequentially as:

$$\begin{array}{c} D\left(x,y\right)=\arg\min_{d\in {\vec{d}}_{p}}\frac{\sum_{u^{x}\in {\vec{u}}_{p}^{x}}\sum_{u^{y}\in {\vec{u}}_{p}^{y}}\omega \left(u^{x},u^{y}\right)\tau \left(u^{x},u^{y},d\right)}{\sum_{u^{x}\in {\vec{u}}_{p}^{x}}\sum_{u^{y}\in {\vec{u}}_{p}^{y}}\omega \left(u^{x},u^{y}\right)} \end{array}$$

where:

$$\begin{array}{ccc} \omega (u^{x},u^{y}) & = & e^{\left(-\frac{\left|\right|I_{r}\left(x,y\right)-I_{r}\left(u^{x},u^{y}\right){\left|\right|}^{2}}{2\sigma_{r}^{2}}\right)}e^{\left(-\frac{{\left(x-u^{x}\right)}^{2}+{\left(y-u^{y}\right)}^{2}}{2\sigma_{d}^{2}}\right)}\\ \tau (u^{x},u^{y},d) & = & min(\lambda \zeta ,|D\left(u^{x},u^{y}\right)-d|) \end{array}$$

where $\sigma_{r}$ and $\sigma_{d}$ are intensity and distance smoothing parameters, respectively (the first factor of $\omega (u^{x},u^{y})$ is the range kernel for smoothing differences in intensities, and the second is the spatial kernel for smoothing differences in coordinates); λ=0.2 is a constant to reject outliers; and *ζ* is a threshold for edges. Notice that the parameter *ζ* is defined for the pixels around depth discontinuities; therefore, each pixel needs to be checked to determine if it is on a depth edge before it is processed. To improve the speed, a GPU implementation was used for this post-processing step.

**Figure 5:**
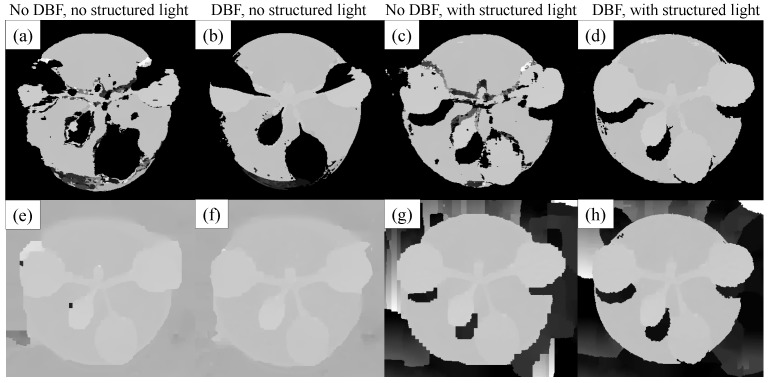
Results of the block matching (BM) (first row) and belief propagation (BP) (second row) algorithms with the use of disparity bilateral filtering (DBF) and structured light (SL): (**a**) BM result without SL; (**b**) BM + DBF result without SL; (**c**) BM result with SL; (**d**) BM + DBF result with SL; (**e**) BP result without structured light (SL); (**f**) BP + DBF result without SL; (**g**) BP result with SL; and (**h**) BP + DBF result with SL. The BM + DBF algorithm with SL is used in our system.

Figure 5 presents stereo matching outcomes, from the example images in Figure 4, of the block matching (BM) and belief propagation (BP) algorithms with the use of the disparity bilateral filter (DBF) and the structured light (SL). This figure shows that structured light can help to improve the matching significantly. The first row of Figure 5 shows the outcome when using BM, and the second row is for BP. The same parameter sets were used for each application of BM and BP. For BM, the number of disparities was 256, the window size was 17, the texture threshold was 60 and a Sobel pre-filter was used. In the case of BP, the number of pyramid levels (for multiscale processing) was four; the number of iterations at each pyramid level was nine; and the number of disparity levels at the first pyramid level was eight. Parameters for DBF were 10 iterations and a filter radius of 41. The first two columns of Figure 5 show matching results without using SL, and the remaining two columns use SL. BM is preferred over BP because of its straightforward implementation, fast processing time and high quality outcome when used with DBF and SL.

### 3.3. Point Cloud Creation from Disparity Values

The final disparity map is converted to a 3D point cloud by calculating the coordinate (X,Y,Z) of each point as:

$$\begin{array}{c} \left[\begin{array}{c} X\\ Y\\ Z \end{array}\right]=\left[\begin{array}{c} x^{\prime }/w^{\prime }\\ y^{\prime }/w^{\prime }\\ z^{\prime }/w^{\prime } \end{array}\right] \end{array}$$

where $x^{\prime }$, $y^{\prime }$, $z^{\prime }$ and $w^{\prime }$ are computed based on the 4 × 4 perspective transformation matrix *Q* (obtained via camera calibration) and the disparity map *D*:

$$\begin{array}{c} \left[\begin{array}{c} x^{\prime }\\ y^{\prime }\\ z^{\prime }\\ w^{\prime } \end{array}\right]=Q\left[\begin{array}{c} x\\ y\\ D(x,y)\\ 1 \end{array}\right] \end{array}$$

**Figure 6:**
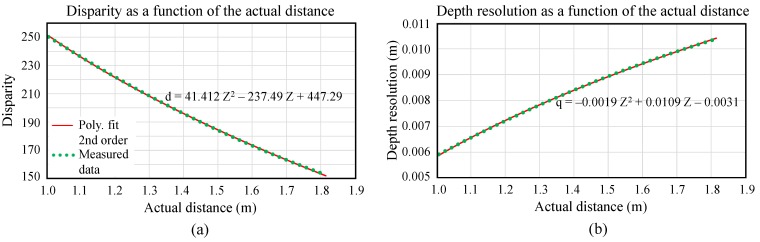
Estimation of the depth resolution of our stereo system as a function of the target distance from 1–1.82 m away from the camera: (**a**) Plot of disparities *versus* actual distances and (**b**) Plot of depth resolutions *versus* actual distances.

The whole plant can be easily extracted in 3D space based on its distance (*Z* values) to the camera. Note that there is a quantization effect in the disparity map, as well as in the 3D point cloud. In our system, the target plant is positioned at approximately 1.4 m from the camera, and its depth resolution (the distance between two successive disparity values) can be estimated using the function:

$$\begin{array}{c} q=-0.0019Z^{2}+0.0109Z-0.0031 \end{array}$$

where *Z* and *q* are in meters. For our case, *q* is approximately 8 mm. This function is built based on manual measurement of disparity values and actual object distances. Figure 6 shows disparity and depth resolution estimated in the stereo system as a function of the actual distance.

### 3.4. Point Cloud Registration

In the usual method, to register two point clouds, the following procedure is sequentially executed on the two clouds: (1) noise removal; (2) 3D keypoint detection; (3) 3D descriptor extraction; (4) 3D correspondence and filtering; (5) initial alignment of the source to the target cloud; and (6) cloud registration [16]. Noise removal is based on 3D cluster extraction, and those clusters having a number of points below a threshold might be discarded. SIFT (scale-invariant feature transform) and estimation of normals are popular in detecting 3D keypoints and extracting their descriptors. Correspondences between two point clouds are then found and filtered using the detected keypoints and descriptors from the previous step. The initial alignment matrix (from the source to the target point cloud) is then calculated using the two keypoint lists and the correspondences. Afterwards, an iterative closest point algorithm (ICP) is used to register the two clouds based on the calculated initial alignment matrix and the predefined parameters of maximum correspondence distance, maximum iterations and outlier rejection threshold.

In our system, camera positions are fixed, with sightlines at 25, 40, 55, 70 and 85 degrees from horizontal, respectively, and aimed at the target object. Hence, there are known relationships between each of the stereo camera pairs, and the five pairs can be calibrated. This leads to an initial alignment matrix that can be estimated directly from the two point clouds created in two stereo camera views. Consequently, the steps of noise removal, keypoint detection, descriptor extraction and correspondence filtering are skipped in this implementation. The method in [37] is used to estimate the transformation of the source to the target point cloud based on four congruent points. By computing the best rigid alignment according to the LCP (largest common point set) measure, this method is effective and robust to noise without the need of pre-filtering or denoising the data. Let $C_{1}$, $C_{2}$, $C_{3}$, $C_{4}$ and $C_{5}$ be the point clouds obtained from the views of 85, 70, 55, 40 and 25 degrees, respectively. The transformation matrices $T_{13}$, $T_{23}$, $T_{43}$ and $T_{53}$ are predefined, where $T_{ij}$ represents the transformation of the source cloud *i* to the target cloud *j*. Then, we have:

$$\begin{array}{ccc} C_{1}^{\prime } & = & T_{13}C_{1}\\ C_{2}^{\prime } & = & T_{23}C_{2}\\ C_{4}^{\prime } & = & T_{43}C_{4}\\ C_{5}^{\prime } & = & T_{53}C_{5} \end{array}$$

where $C_{i}^{\prime }$ is a transformed cloud of $C_{i}$ according to $T_{ij}$. An ICP algorithm is executed for each pair of $C_{1}^{\prime }$ and $C_{3}$, $C_{2}^{\prime }$ and $C_{3}$, $C_{4}^{\prime }$ and $C_{3}$ and $C_{5}^{\prime }$ and $C_{3}$, respectively, to optimize the overlap between the each pair of clouds:

$$\begin{array}{ccc} C_{1}^{\prime \prime } & = & C_{1}^{\prime }⊕C_{3}\\ C_{2}^{\prime \prime } & = & C_{2}^{\prime }⊕C_{3}\\ C_{4}^{\prime \prime } & = & C_{4}^{\prime }⊕C_{3}\\ C_{5}^{\prime \prime } & = & C_{5}^{\prime }⊕C_{3} \end{array}$$

where ⊕ represents the registration using ICP. Lastly, the final registered point cloud is the merger of the clouds $C_{1}^{\prime \prime }$, $C_{2}^{\prime \prime }$, $C_{3}$, $C_{4}^{\prime \prime }$, $C_{5}^{\prime \prime }$:

$$\begin{array}{c} \text{C}=C_{1}^{\prime \prime }+C_{2}^{\prime \prime }+C_{3}+C_{4}^{\prime \prime }+C_{5}^{\prime \prime } \end{array}$$

where + is a merging operator. Notice that ICP is ill-conditioned for small, quantized, near-planar point clouds when it is executed alone. By doing the LCP-based method and then ICP, registration of two clouds does not meet the ICP ill-condition and is optimized for the largest overlap between the clouds. Optional steps can be done after having *C*, such as noise removal based on clustering and voxel grid conversion to reduce the number of points in the cloud. These steps help optimize processing speed and simplify the output for the extraction of 3D features. Transformations of the clouds obtained in different turn-table angles (resolution of 45 degrees) are also precomputed and optimized in the same manner.

**Figure 7:**
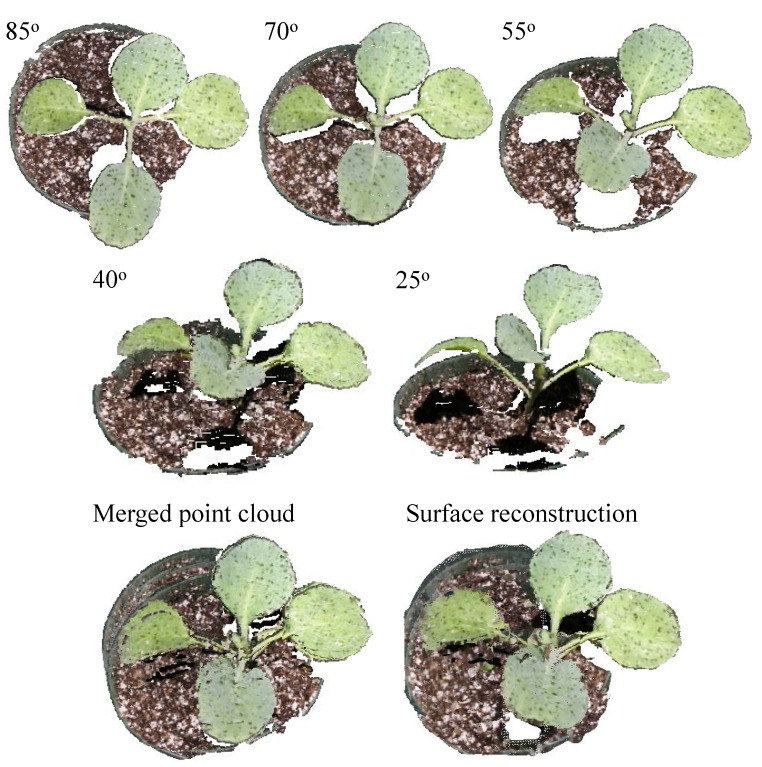
Point clouds from different view angles (top row) and their merged point cloud and surface reconstruction results (bottom row).

Due to the quantization effect, two successive disparity levels of the point cloud obtained in this system at a distance of 1.4 m have a resolution of approximately 8 mm. Thus, surface reconstruction is needed to smooth and reform the point cloud to fix the quantization problem. Poisson surface reconstruction is utilized to improve the accuracy of plant feature measurement. Figure 7 shows the sample results of the merged point cloud and surface reconstruction, where five point clouds at different camera view angles are used as the input. To get the results shown in Figure 7, the original result of the Poisson surface reconstruction has to go through superfluous vertex/face removal steps: conditional vertex/face selection, faces-having-long-edges selection and isolated face removal.

### 3.5. 3D Feature Extraction of a Plant

This section describes the algorithms used for estimating plant height, the number of leaves, leaf size and internode distances. Before feature extraction, the reconstructed point cloud is processed using a clustering method to divide the unorganized cloud into smaller parts so that plant features can be recognized effectively. The Euclidean clustering method is utilized by making use of 3D grid subdivision with fixed width boxes [38].

**Figure 8:**
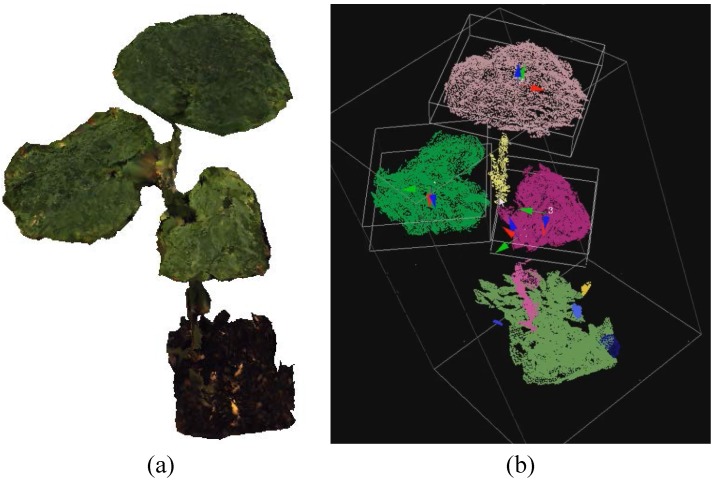
3D model of a cucumber plant (**a**) and its leaf detection result (**b**), where each part of the plant is color-coded and the bounding boxes of leaves are shown.

**Figure 9:**
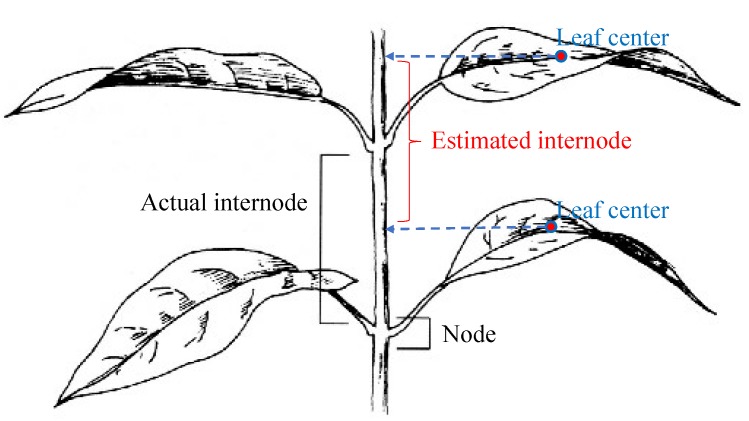
Illustration of internode distance estimation, where leaf centers are projected onto the plant’s principal axis. (The original internode image is from http://pixgood.com/internode-plant.html).

- Plant height: Plant height was defined as the absolute difference in the *Z* direction between the minimum and maximum 3D coordinates within the whole point cloud.
- Number of leaves/leaflets (leaf detection): To enumerate the number of leaves/leaflets, a cluster is considered a leaf/leaflet when these three Boolean conditions are satisfied:
  $$\begin{array}{ccc} C_{size} & = & \left(Eval_{max}>Thr_{size}\right)\\ C_{direction} & = & \left(\frac{Eval_{max}}{Eval_{sum}}<Thr_{direction}\right)\\ C_{position} & = & \left(Centroid>Ratio\times PlantHeight\right) \end{array}$$
  where $Eval_{max}$ is the maximum eigenvalue of the cluster; $Eval_{sum}$ is the sum of all eigenvalues; $Thr_{size}$ and $Thr_{direction}$ are thresholds for the conditions $C_{size}$ and $C_{direction}$, respectively; Ratio is a number defining locations for leaves; and $C_{direction}$ implies that the magnitude of the dominant direction should not be much larger than that of other directions. Figure 8 shows a leaf detection result of a three-leaf cucumber plant, where its cloud is color-coded and displayed with bounding boxes using the Point Cloud Library [16].

- Leaf size: Based on the detected leaves, a bounding box for each leaf is determined by using the leaf eigenvectors and centroid. The product of the width and length of the bounding box is defined as the leaf size.
- Internode distance: The internode distance is the distance between two leaf nodes along a plant stem. Detecting nodes in a 3D point cloud is not a simple task; therefore, the following method is used to estimate the internode distances: leaf centers are projected onto the principal axis of the whole plant, then the distance between these projected points is considered as the internode distance. Figure 9 illustrates this method of internode distance estimation.

## 4. Experimental Results

**Figure 10:**
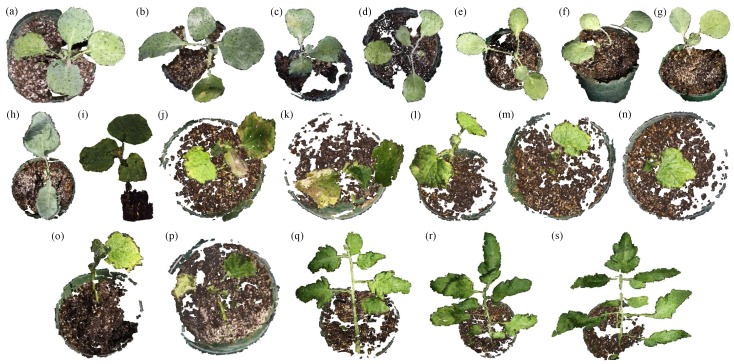
3D model results of eight cabbage plants (**a**–**h**), eight cucumber plants (**i**–**p**) and three compound leaves from tomato plants (**q**–**s**).

A computer (CPU Model Core i7 at 3.4 GHz, Intel Co., Santa Clara, CA, USA, with 12-GB DDR3 random-access-memory) was used for all processing steps, except that a 1152-core GPU (Model GeForce GTX 760, NVidia Co., Santa Clara, CA, USA) graphics card was utilized for implementing the GPU-based stereo matching algorithms. Experiments were executed on eight cabbage plants, eight cucumber plants and three tomato compound leaves. These plant species were selected as examples of plants with: long leaves, leaves spreading vertically, very small leaves, curved leaves, long branches, overlapped leaves, leaves having natural texture (Figure 10j,k,p) and compound leaves with leaflets attached to a rachis. The ground truth plant heights were measured manually based on the distance from the plant’s base to the top point of the highest leaf. In the same manner, the ground truth internode distances were manually determined based on the distance between two leaf nodes along a plant stem, as illustrated in Figure 9. The ground truth leaf sizes were measured by the steps of cutting off the leaves, scanning their images and then creating a ruler-based mapping from a pixel unit to a real-world unit (mm) for the leaves. Leaf detection accuracy and errors in estimating the plant height, leaf size and internode distance were quantified. Figure 10 shows the reconstructed 3D models of the 19 plants. Table 1 presents a brief description of the plants consisting of plant height and number of leaves. In this experiment, at least five views (at one turn-table angle) were needed to successfully reconstruct a whole plant. The cucumber (Figure 10i) and the three tomato compound leaves (Figure 10q–s) required 15 views (five views at a turn-table angle × three turn-table angles with a difference of 45 degrees) because of the high degree of self-overlap and curve-shaped leaves in these species.

**Table 1:** List of the 19 plants used for the experiments.

Plant	Figure 10	Height ${}^{+}$	No. of Leaves	Brief Description
Cabbage 1	(a)	114	4	Good leaf shape
Cabbage 2	(b)	150	4	1 vertically-long leaf
Cabbage 3	(c)	140	4	1 small and 2 curved leaves
Cabbage 4	(d)	114	4	Long branches
Cabbage 5	(e)	130	4	2 overlapped leaves
Cabbage 6	(f)	139	3	Long and thin branches
Cabbage 7	(g)	105	3	1 leaf attaches to plant stem
Cabbage 8	(h)	229	2	1 curved leaf
Cucumber 1	(i)	242	3	Tall, big leaves
Cucumber 2	(j)	117	4	2 brown-textured-surface leaves
Cucumber 3	(k)	131	3	2 brown-textured-surface leaves
Cucumber 4	(l)	115	2	1 small leaf
Cucumber 5	(m)	113	1	Good leaf shape
Cucumber 6	(n)	123	2	1 small leaf
Cucumber 7	(o)	132	2	1 leaf attaches to plant stem
Cucumber 8	(p)	116	2	1 yellow-textured-surface leaf
Tomato 1	(q)	192 *	6	Long and curved leaves
Tomato 2	(r)	253 *	8	Long and curved leaves
Tomato 3	(s)	269 *	8	Long and curved leaves

^+^ Unit in mm; * length of the compound leaf.

In this system, the parameters of the mechanical structure were fixed, while parameters for the software algorithms were adapted to optimize the performance in phenotyping different species. Table 2 shows the parameters used for the GPU-based stereo matching and point cloud registration to 3D feature extraction steps. This system required parameter tuning so that high quality results can be obtained. With the selected parameters used in the segmentation step, each plant was completely separated from the background, which helps to improve the stereo matching results significantly. The actual distance of the target plant to the cameras was chosen from 1.2 m–1.7 m, so that all data points outside that range (*i.e.*, background noise) were discarded when reprojecting disparity values onto 3D space. In the feature extraction step, individual parameters, such as clustering tolerance, minimum cluster size, leaf size threshold, leaf direction threshold and ratio for leaf location, might be adjusted for each point cloud in order to correctly detect all leaves.

**Table 2:** Algorithm parameters used for the experiments.

GPU-Based Stereo Matching				Point Cloud Registration				3D Feature Extraction *		
Plant segmentation	Spatial win-size	11		Registration	Max distance	25		Clustering	Tolerance	0.03
	Color win-size	7			Max iteration	10${}^{3}$			Min cluster size	4000
	Min segment size	10			Outlier rejection	25			Max cluster size	10${}^{5}$
	Threshold	10		Poisson surface reconstruction	Octree depth	12		Leaf detection	Size threshold	0.005
Stereo block matching	No. of disparities	256			Solver divide	7			Direction threshold	0.7
	Win-size	17			Samples/node	1			Ratio: leaf location	0.25
	Texture threshold	60			Surface offset	1		* Parameters vary depending on leaf shape		
Bilateral filter	Filter size	41		Face removal w.r.t. edge length		0.05				
	No. of iterations	10		Noise removal w.r.t. No. of faces		25				

**Figure 11:**
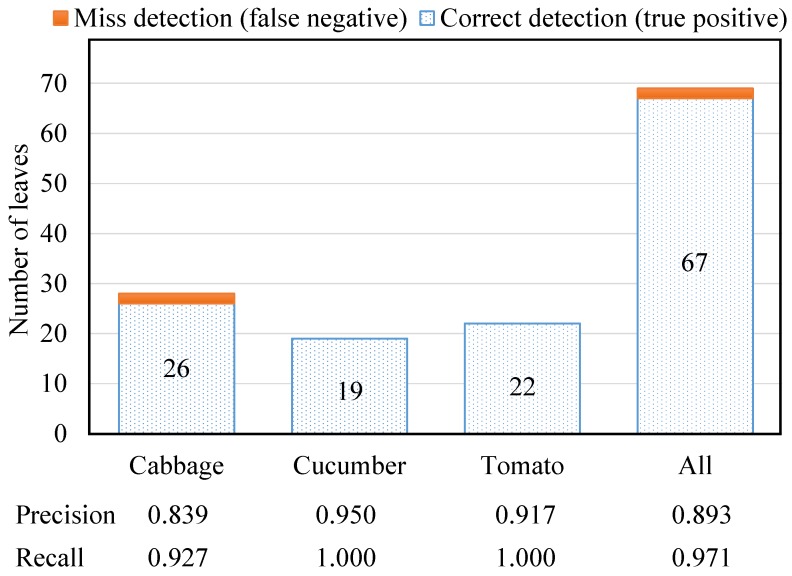
Evaluation of the number of detected leaves/leaflets in terms of precision and recall, from the 3D reconstructed cabbage, cucumber and tomato plants.

**Figure 12:**
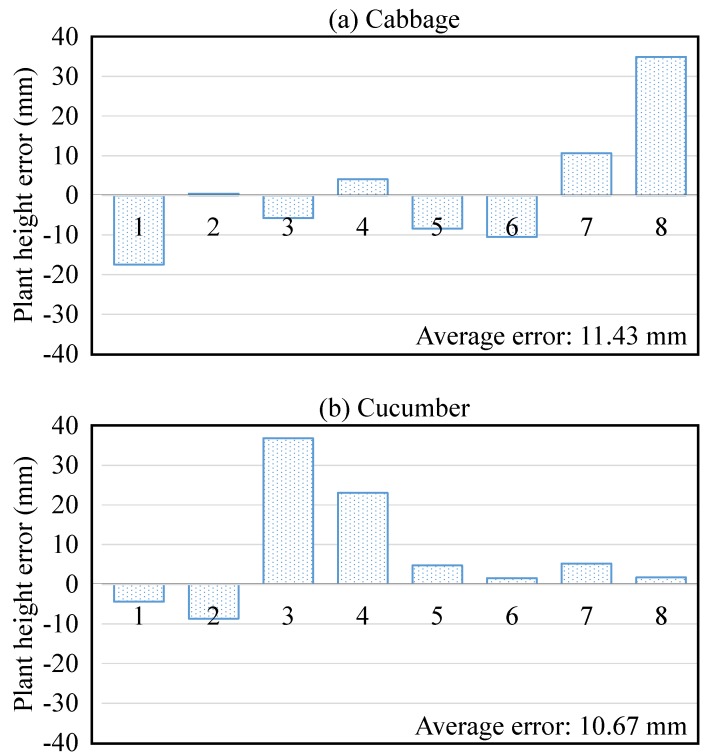
Errors of plant height (calculated by differentiating the ground truth from the estimated one) of the cabbage (**a**) and cucumber plants (**b**). Notice that plant height was not determined for tomato compound leaves, because these leaves were imaged individually and are parts of a plant.

Table 3 presents average measurement accuracies in plant phenotype estimation using the features extracted from 3D reconstruction when using the structured illumination system. Figure 11 and Figure 12 show leaf detection accuracies (in terms of precision and recall) and plant height errors accordingly. In Figure 11, the precision and recall were computed based on the number of correct detections (true positive), the number of incorrect detections (false positive) and the number of missed detections (false negative) to show the robustness of the detection. The leaf/leaflet detection accuracies were 93.75%, 100% and 100% for cabbage, cucumber and tomato, respectively. The main source of error in estimating the phenotyping measurements in cabbage was because the cabbage plants had smaller leaves than the cucumber and tomato. Additionally, cabbage leaves had greater curvature and vertical spread. Positive and negative errors were presented in Figure 12 to explain that the actual plant height was mostly larger than the estimated height. The average plant height error for all plants is 11.18 mm. The percentage of error in Table 3 was computed as a normalized value, so that we could directly compare the results between different types of plants. The average error in estimating leaf and internode features, as a percentage of plant height across all three species was, 4.87%, 3.76% and 7.28% for leaf length, width and internode distance, respectively. The main reason for the higher internode distance error was that this distance was estimated based on the plant’s principal axis (as aforementioned in Figure 9), which was approximated by the most dominant eigenvector of the plant. The approximation of the principal axis by the dominant eigenvector was somewhat inaccurate when leaves or branches of the plant spread unexpectedly in different directions.

**Table 3:** Average accuracy in plant phenotype estimation from 3D reconstruction.

Plant Features		Cabbage	Cucumber	Tomato	Average
Leaf height	Error (mm)	6.86	5.08	10.16	6.6
	% error *	5.58%	4.36%	4.36%	4.87%
Leaf width	Error (mm)	5.08	4.83	5.33	5.08
	% error *	4.16%	3.9%	2.31%	3.76%
Internode distance	Error (mm)	9.65	7.87	21.34	10.92
	% error *	7.67%	6.3%	8.49%	7.28%

* Percentage of error over plant height.

**Figure 13:**
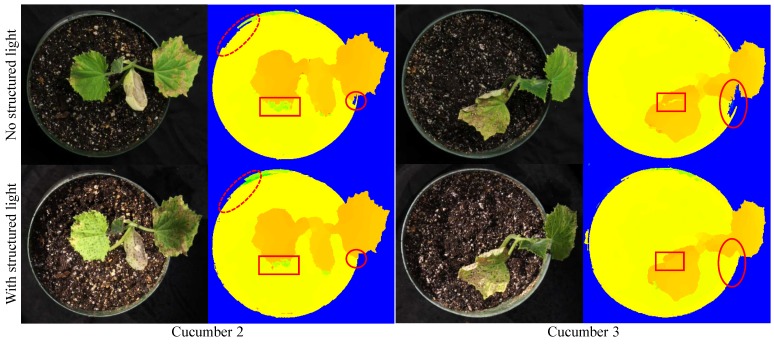
Comparison between without and with using structured light (SL) on textured leaves. The disparity result of using SL is slightly better than that without using SL in the regions of less natural textures (marked by red rectangles and ellipses). Note that the colorized disparity images are presented here, instead of grayscale ones, for better illustration of the differences.

Without structured light, many of the leaves of the plants in this study could not be matched correctly using the block matching algorithm, as mentioned in Figure 5a,b, illustrating the benefit of enhancing the visual texture of plants using structured illumination. In some cases, the leaves had sufficient natural texture to allow successful stereo matching, as in Cucumber 2, 3 and 7. However, sometimes, the natural texture was actually a defect, like a scar or insect damage on the leaf, that created the texture and was not a feature of the plant, but of the environment. The system generally had to work with leaves where the plants were healthy and had less leaf texture. A comparison of the algorithm performance between plant images without and with using structured light to enhance the leaf texture is shown in Figure 13. The disparity result obtained when using structured light was slightly better than that obtained without using structured light in the leaf areas with less natural texture.

**Figure 14:**
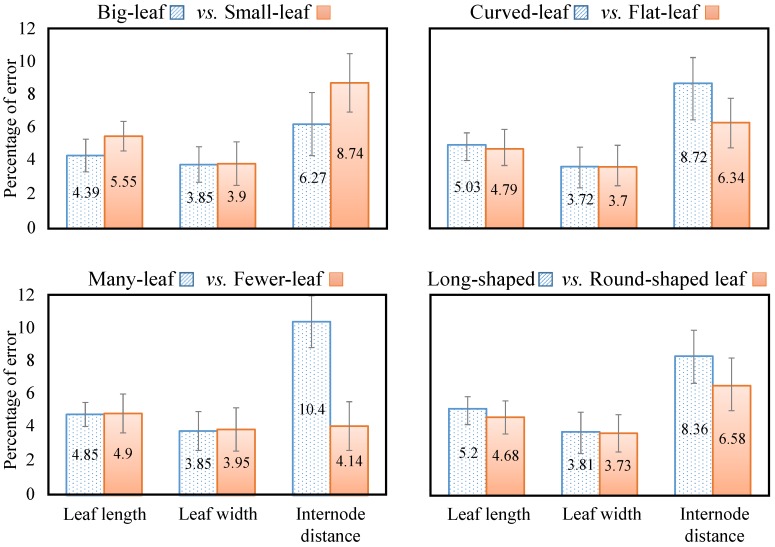
Comparison of the percentage of error in plant height estimation for plants having different leaf sizes, leaf shapes and numbers of leaves. Errors for leaf length, leaf width and internode distance were considered in order to understand which types of plant leaves yield higher errors. From left to right, top to bottom: plants having big leaves *versus* plants having small leaves, plants having curved *versus* flat leaves, plants having many *versus* fewer leaves and plants having long-shaped *versus* round-shaped leaves.

A comparison of the system performance for plants having different leaf sizes, leaf shapes and number of leaves was conducted. Figure 14 shows the results for plants having big leaves *versus* plants having small leaves, plants having curved leaves *versus* flat leaves, plants having many leaves *versus* plants having fewer leaves and plants having long-shaped leaves *versus* plants having round-shaped leaves. When comparing leaf size, we found that larger leaves are easier to detect than the small leaves. The main reason for the superior detection of larger leaves is that, at the image resolution of 1920 × 1280 pixels, leaves having a size of 40 mm or less at a distance of more than 1.2 m were tiny objects in the image, and they might give more errors in the stereo matching (with a predefined matching window size for all kinds of leaves in a plant) and point cloud registration (clouds having more points were easier for registration and reconstruction) steps. The internode distance error for the plants having small leaves was significantly higher than for plants having big leaves. Estimation of internode distances was affected by calculation of a plant’s principal axis. Big leaves spreading unexpectedly in different directions might make the plant’s principal axis be appear at a different position than the plant’s stem; therefore, it yielded higher errors in the estimation of internode distances. Leaf size errors were approximately the same in the cases of “curved-leaf *vs.* flat-leaf”, “many-leaf *vs.* fewer-leaf” and “long-shaped *vs.* round-shaped leaf”; indicating that leaf shape and the number of leaves did not actually affect the estimation. Note that in the system, a plant was imaged from many different views in order to have the full shape of the leaves in 3D; thus, it was presumed that acceptable 3D models of leaves were used for the feature extraction step. However, internode distance errors were notably different for various leaf sizes, leaf shapes and numbers of leaves. Curved and long leaves yielded high errors in the internode distance estimation. Plants having fewer leaves gave highly accurate internode evaluation. Vertical error bars in Figure 14 confirm the significant differences between the internode distance errors in terms of leaf size, leaf shape and the number of leaves.

In the present practice, plant phenotype features are manually measured by destroying the plant, with high human time consumption. As reported in [39], it normally took more than 2 h for two people to destructively measure the total leaf area of a single row of some large pepper plants. The study [19] stated that manual phenotypic analysis required approximately 30 min per plant. It took an average of 20 min per plant for manual measurement of our experimental plants. Our system, which is non-destructive, fully utilized C++ and CUDA C languages to create a 3D model of a plant from five stereo image pairs. It required approximately 4 min of processing time in total, which includes: less than 4 s (1.5% of the total time) for transferring the images from the ten cameras via the USB to the computer (camera shutter speed is 250 ms), approximately 5 s (2%) for plant segmentation, 0.3 s for stereo matching, less than 4 s (1.5%) for disparity bilateral filtering, 0.08 s for reprojection of disparity values to the 3D cloud, 180 s (75%) for cloud registration (variable depending on the number of data points and complexity of the transformation between the source and target point clouds), approximately 40 s (17%) for Poisson surface reconstruction and less than a second for leaf detection and 3D feature extraction. By utilizing a GPU, plant segmentation was approximately 20-times faster than the CPU-based implementation [30]. The processing time of the block matching and disparity bilateral filtering was approximately improved by 30- and 10-times [36], respectively. We plan to implement the steps of point cloud registration and processing on a GPU for future deployment of the system, so that a total time of approximately 1 min for a complete 3D plant model is expected.

It was not possible to directly compare the existing systems to ours where different plant features, cameras and software configurations were used. Table 4 summarizes and compares various camera-based 3D reconstruction systems for plants in terms of their system configuration, features, methods, accuracy and processing speed. Comparing and analyzing such systems enabled us to highlight their advantages and disadvantages. Additionally, it allowed us to relate the performance of our system to the existing ones via the percentage of absolute errors and total processing time. In terms of leaf detection, our system yielded a substantially better accuracy (97%) than that of [18] (68%). Our plant height error, 8.1%, was smaller than the 9.34% of [19]. The leaf width and height errors obtained from our system, 3.76% and 4.78%, greatly outperformed the 5.75% and 8.78% errors of [19]. Our full 3D reconstruction system required approximately 4 min for the whole process from image capturing to plant feature extraction in comparison to 20 min of [19] and 48 min of [22], respectively, where commercial 3D modeling software were utilized. It took a minute for full 3D leaf segmentation and modeling in [13]. In a single view, the systems in [14,39] required more than an hour and 5 min respectively, to process a large plant.

**Table 4:** Comparison of various camera-based 3D reconstruction systems for plants.

Study	Camera System	Camera View	Measures	Environment	Techniques	Accuracy	Processing Time
Alenya, 2011 [13]	ToF and color cameras; robot arm	Multiview for leaf modeling	Leaf size	Indoor	Depth-aided color segmentation, quadratic surface fitting, leaf localization	Square fitting error: 2 cm^2^	1 min for 3D leaf segmentation
Chene, 2012 [18]	Kinect camera	Top view	Leaf azimuth	Indoor	Maximally stable extremal regions-based leaf segmentation	Detection accuracy 68%; azimuth error 5%	n/a
Heijden, 2012 [39]	ToF and color cameras	Single view	Leaf size and angle	Greenhouse (for large pepper plants)	Edge-based leaf detection, locally weighted scatterplot smoothing-based surface reconstruction	Leaf height correlation 0.93; leaf area correlation 0.83	3 min for image recording, hours for the whole process
Paproki, 2012 [19]	High-resolution SLR camera, with 3D modeling software [20]	Multiview for full 3D reconstruction	Plant height, leaf width and length	Indoor	Constrained region growing, tubular shape fitting-based stem segmentation, planar-symmetry and normal clustering-based leaf segmentation, pair-wise matching-based temporal analysis	Plant height error 9.34%; leaf width error 5.75%; leaf length error 8.78%	15 min for 3D reconstruction [20], 4.9 min for 3D mesh processing and plant feature analysis
Azzari, 2013 [15]	Kinect camera	Top view	Plant height, base diameter	Outdoor	Canopy structure extraction	Correlation: 0.97	n/a
Ni, 2014 [12]	2 low-resolution stereo cameras with 3D modeling software [7]	Multiview for full 3D reconstruction	Plant height and volume, leaf area	Indoor	Utilizing VisualSFM software [7], utilizing [40] to manually extract plant features	n/a	n/a
Song, 2014 [14]	Two stereo cameras; ToF camera	Single view	Leaf area (foreground leaves only)	Greenhouse (for large plants)	Dense stereo with localized search, edge-based leaf detection, locally weighted scatterplot smoothing-based surface reconstruction	Error: 9.3%	5 min for the whole process
Polder, 2014 [23]	3D light-field camera	Single view	Leaf and fruit detection	Greenhouse (for large tomato plants)	Utilizing 3D light-field camera to output a pixel to pixel registered color image and depth map	n/a	n/a
Rose, 2015 [22]	High-resolution SLR camera, with 3D modeling software [41]	Multiview for full 3D reconstruction	Plant height, leaf area, convex hull	Indoor	Utilizing Pix4Dmapper software [41] to have 3D models, plant feature extraction	Correlation: 0.96	3 min for data acquisition, 20 min for point cloud generation, 5 min for manual scaling, 10 min for error removal
Andujar, 2015 [21]	4 Kinect cameras with 3D modeling software [42]	Multiview for semi-full 3D reconstruction	Plant height, leaf area, biomass	Outdoor	Utilizing Skanect software [42] to have 3D models, utilizing [40] to manually extract plant features	Correlation: plant height 0.99, leaf area 0.92, biomass 0.88	n/a
Our system	10 high-resolution SLR cameras organized into 5 stereo pairs; 2 structured lights	Multiview for full 3D reconstruction	Plant height, leaf width and length, internode distance	Indoor	Texture creation using structured lights, mean shift-based plant segmentation, stereo block matching, disparity bilateral filtering, ICP-based point cloud registration, Poisson surface reconstruction, plant feature extraction	Leaf detection accuracy 97%; plant height error 8.1%, leaf width error 3.76%, leaf length error 4.87%, internode distance error 7.28%	4 min for the whole process

## 5. Conclusions and Future Work

A multi-stereo digital imaging system for 3D virtual reconstruction of whole plants at the macro level was successfully designed, fabricated and tested. The system uses ten high-resolution color digital cameras set up as five pairs of stereo imagers, mounted on an arc superstructure designed for field deployment. A custom-designed structured light pattern illumination system was developed to improve the performance of the software algorithms and to obtain better 3D models and more accurate phenotyping measurements.

The ability to produce 3D models of whole plants created from multiple pairs of stereo images captured at different angles of view, without the need to destructively cut off any parts of a plant, was demonstrated. These 3D models allowed phenotyping features, such as the number of leaves, plant height, leaf size and internode distances, to be estimated. For plants having an appropriate leaf size of greater than 38 mm and a distance between leaves greater than 50 mm, the algorithms work successfully with an accuracy of more than 97% for leaf detection and less than a 13-mm error for estimating plant and leaf size and internode distance.

Future work should include: (1) automating and synchronizing the cameras and structured lights, so that the system can be deployed in the field; (2) adding more structured lights and increasing their projection range to help in reconstructing larger and more complicated plants; and (3) optimizing the algorithm parameters to support a wider range of plant species with less parameter tuning.
